# Part 2: Adaptation of Gait Kinematics in Unilateral Cerebral Palsy Demonstrates Preserved Independent Neural Control of Each Limb

**DOI:** 10.3389/fnhum.2017.00050

**Published:** 2017-02-13

**Authors:** Thomas C. Bulea, Christopher J. Stanley, Diane L. Damiano

**Affiliations:** Functional and Applied Biomechanics Section, Rehabilitation Medicine Department, National Institutes of Health, BethesdaMD, USA

**Keywords:** neural circuits, after-effects, brain injury, knee angle, local dynamic stability, neurorehabilitation

## Abstract

Motor adaptation, or alteration of neural control in response to a perturbation, is a potential mechanism to facilitate motor learning for rehabilitation. Central nervous system deficits are known to affect locomotor adaptation; yet we demonstrated that similar to adults following stroke, children with unilateral brain injuries can adapt step length in response to unilateral leg weighting. Here, we extend our analysis to explore kinematic strategies underlying step length adaptation and utilize dynamical systems approaches to elucidate how neural control may differ in those with hemiplegic CP across legs and compared to typically developing controls. Ten participants with hemiplegic CP and ten age-matched controls participated in this study. Knee and hip joint kinematics were analyzed during unilateral weighting of each leg in treadmill walking to assess adaptation and presence and persistence of after-effects. Peak joint angle displacement was used to represent changes in joint angles during walking. We examined baseline and task-specific variability and local dynamic stability to evaluate neuromuscular control across groups and legs. In contrast to controls, children with unilateral CP had asymmetries in joint angle variability and local dynamic stability at baseline, showing increased variability and reduced stability in the dominant limb. Kinematic variability increased and local stability decreased during weighting of ipsilateral and contralateral limbs in both groups compared to baseline. After weight removal both measures returned to baseline. Analogous to the temporal-spatial results, children with unilateral CP demonstrated similar capability as controls to adapt kinematics to unilateral leg weighting, however, the group with CP differed across sides after weight removal with dominant limb after-effects fading more quickly than in controls. The change in kinematics did not completely return to baseline in the non-dominant limb of the CP group, producing a transient improvement in joint angle symmetry. Recent studies demonstrate that neural control of gait is multi-layered with distinct circuits for different types of walking and for each leg. Remarkably, our results demonstrate that children with unilateral brain injury retain these separate circuits for each leg during walking and, importantly, that those networks can be adapted independently from one another to improve symmetry in the short term.

## Introduction

Motor adaptation is defined as a gradual, trial-by-trial modification of a previously learned movement in response to a specifically applied perturbation that is retained, at least briefly, as an after-effect following its removal (Martin et al., 1996; Bastian, 2008). The distinguishing feature of motor adaptation is the after-effect, which has been postulated as evidence that the perturbation altered the internal dynamical model used by the central nervous system to control movement (Shadmehr and Mussa-Ivaldi, 1994). The possibility of altering neural control makes adaptation to a perturbation an attractive potential mechanism for motor learning strategies to enhance skill level during training and rehabilitation.

Because of its importance for independence and daily living, the ability to modify locomotor strategies via motor adaptation has been studied extensively. Split belt treadmill based paradigms, in which one belt is sped up while the other is slowed down for a pre-determined time interval and then returned to the same speed, are commonly used to study adaptation during locomotion; for a review see Torres-Oviedo et al. (2011). In healthy adults, split belt paradigms have consistently shown that interlimb parameters which involve both legs, such as double support time, swing time, and stride length, show adaptation with strong after-effects (Dietz et al., 1994; Reisman et al., 2005), while intralimb parameters, such as limb excursion and timing of joint kinematics, showed either an immediate or no change with no after-effect, suggesting no adaptation occurred (Reisman et al., 2005). Others have examined weighting of a single limb in healthy adults during walking, which showed immediate changes in bilateral kinematics followed by slow adaptation back toward baseline levels (Noble and Prentice, 2006; Savin et al., 2010). After removal of the weight, kinematics showed an overshoot following by a quick return to baseline levels, i.e., an after-effect. The same effect on lower extremity joint kinematics has also been demonstrated using a velocity-dependent force field paradigm in a robotic gait trainer (Cajigas et al., 2010).

Deficits in the central nervous system are known to affect locomotor adaptation capability. In particular, cerebellar damage has been shown to affect interlimb adaptation in a split belt paradigm while immediate non-adaptive intralimb changes were unaffected (Morton and Bastian, 2006). Changes in adaptation capability may be related to injury severity as those with mild cerebellar ataxia some show similar adaptation as healthy controls (Hoogkamer et al., 2015). Interestingly, in a separate study, those with cerebellar damage showed more variability in intralimb coordination in response to limb weighting compared to healthy controls (Ilg et al., 2008), suggesting adaption strategy and the structures involved may be perturbation specific. The ability of those with unilateral stroke to adapt step symmetry and other interlimb parameters to a split-belt treadmill suggests that the cerebellum, not cerebral structures, is primarily responsible for motor adaptation (Reisman et al., 2010; Malone and Bastian, 2014).

Brain development may also play a role in the capacity for adaptation. A split belt treadmill study of children less than 3 years of age found that adaptation was inconsistently observed within the group, with 23/26 children showing adaptation of double support time with after-effects, while only 12/27 showed adaptation in step length (Musselman et al., 2011). These results agree with another study showing that children younger than 6 years did not adapt spatiotemporal parameters in a split belt paradigm while children as old as 11 took longer to adapt and for after-effects to disappear than healthy adults (Vasudevan et al., 2011).

Repeated adaptation and de-adaptation may result in motor learning, or the formation of a new motor pattern through long-term practice (Bastian, 2008). Such motor learning could be effective for rehabilitation, though that remains an open question. Preliminary evidence suggests that beneficial effects of short-term adaptation on step symmetry in those with stroke may be extended by repetitive training (Reisman et al., 2013), although the effect was only seen in roughly half of the participants and may have been related to baseline characteristics. Similarly, weighting of both the paretic (Lam et al., 2009) and non-paretic (Regnaux et al., 2008) leg appears to have training benefits on functional ability after weight removal.

In our analysis of the temporal-spatial results from the same cohort as reported here, we demonstrated that children with hemiplegic cerebral palsy (CP) have the same capability as age-matched controls to adapt step length in response to unilateral limb weighting (Damiano et al., 2017). These results from our cohort which had 9/10 individuals with focal, unilateral lesions are similar to those from adult onset stroke described above, and provide further evidence that cerebral damage may not be as detrimental to impaired adaptation as damage to the cerebellum.

Yet, much is still unknown about the underlying control of gait and the process of error-based motor learning as it pertains to walking. One difficulty is the multi-layered neural architecture subserving locomotion, which constitutes a hierarchical system that must coordinate between circuits spanning multiple anatomical levels from the spinal cord to the cortex to achieve functional walking. For instance, differences in adaptation of interlimb parameters (e.g., step length) and intralimb parameters (e.g., joint kinematics) to the same perturbation suggests multiple levels of control. Previous studies of healthy individuals have indicated the existence of distinct control networks for different types of walking and for each leg (Choi and Bastian, 2007). Furthermore, variability in the level of conscious attention paid to the perturbation affects spatial and temporal elements of walking differently (Malone and Bastian, 2010) providing additional evidence of multiple, distinct neural circuits underlying control of walking.

Recent models and experimental results demonstrate that short-term motor learning involves a combination of processes operating at both fast- and slow-time scales (Smith et al., 2006), the former of which may be based on recalibration of internal models while the latter may focus on reinforcement of these changes (Huang et al., 2011). Reinforcement theory also suggests that in reward-based motor learning, in which error signals are omitted but feedback is provided based on successful task execution, motor exploration via enhanced movement variability plays a critical role (Izawa and Shadmehr, 2011). Interestingly, recent studies have demonstrated that motor variability is also advantageous for error-based (i.e., perturbation-based) adaptation capability as individuals with higher task-oriented variability at baseline showed faster learning rates (Wu et al., 2014). The same study also demonstrated that, when trained in environments specific to single perturbation type, expedient learners reorganized their output motor variability in line with the perturbation. Thus, motor variability, rather than necessarily reflecting noise in the system output, also appears to play a key role in one’s ability to adapt previously refined motor skills in response to perturbations.

In an interesting parallel, stride-to-stride kinematic variability during walking was traditionally assumed to arise primarily from noise in the motor output of the system, with low deviations interpreted as indicators of a well learned pattern (Winter, 1984). As such, typical gait analysis comparing healthy and pathological walking is based on averaging of data across strides (Winter, 1989). While revealing important quantitative and descriptive measures of activity patterns underlying gait, this approach ignores the overall dynamical nature of walking that may be contained in the variability. Techniques used to study non-linear dynamical systems, such as local dynamical stability, provide further insights into the underlying neuromuscular control of walking (Hurmuzlu and Basdogan, 1994). Local dynamic stability quantifies the system sensitivity to small perturbations such as the natural stride-to-stride variability in joint angles. This approach has been previously deployed to demonstrate that individuals with neuropathic pain reduce gait speed to enhance stability while simultaneously increasing stride-to-stride kinematic variability (Dingwell and Cusumano, 2000), and that motor control strategies optimizing stability during gait are altered in those with unilateral trans-tibial amputations (Wurdeman et al., 2013). Examination of stride-to-stride variability, particularly in the context of local dynamic stability, may provide further insights into underlying shifts in neuromuscular control during adaptation and persistence of aftereffects.

Neuromuscular control differs in children with CP compared to those with typical development (TD). Children with CP exhibit reduced activation during maximum voluntary contraction along with reduced ability to modulate firing rates of some motor units (Rose and McGill, 2005). In submaximal isometric contractions, children with CP have increased variability, especially at more distal joints (Arpin et al., 2013). During walking, children with CP show elevated co-contraction and different activation timing compared to TD (Unnithan et al., 1996). Fewer muscle synergies are required to describe variations in muscle activity during walking in some individuals with CP compared to TD and this reduction has been correlated with clinical measures of function (Steele et al., 2015), suggesting that those with CP utilize less complex control strategies. However, the neuromuscular control complexity during walking in those with CP is still an open research question, and adaptation paradigms offer one method for its study.

The goal of this study was to extend our analyses of temporal spatial data by examining adaptation of intralimb kinematics in response to unilateral leg weighting in children with hemiplegia from CP and age-matched controls. Based on our first study that showed children with CP can adapt step length in response to limb weighting (Damiano et al., 2017), we hypothesized that at the group level children with unilateral CP would show asymmetries between dominant and non-dominant limbs in both kinematic variability and local dynamic stability while typically developing controls would not. We also hypothesized that similar to controls, the group with CP would adapt the kinematics of each limb when weighted. Finally, we expected the adaptation and after-effects to be asymmetric in the CP group demonstrating that similar to healthy individuals, children with unilateral brain injury maintain distinct neural circuitry for controlling each limb.

## Materials and Methods

### Participants

Ten participants with CP (mean age: 14.8 ± 3.8 years, GMFCS range: I–II) were recruited to participate in the study, and 10 participants without CP (mean age: 11.4 ± 3.6 years) were selected from a recruitment database in order to match gender and age to the group with CP. Further details on subject demographics are available in Damiano et al. (2017). All participants met the following inclusion criteria: at least 5 years of age, no surgery within the previous year, no leg injury that may affect their ability to walk, and weight less than 150 lbs. We pre-determined that maximal ankle weight should not exceed 12 lbs., so we further limited participants to 150 lbs. to ensure that the load would remain between 8 and 10% of the participants’ body weight.

This study was approved by the institutional review board (Protocol #90-CC-0168). Written informed consent was obtained from participants 18 years and older. Written consent was obtained from a parent or legal guardian as well as written assent of the participant if they were less than 18 years of age.

### Procedures

Reflective markers were placed on the pelvis and lower extremities, and 3D motion was tracked by a 10-camera MX motion capture system and collected in Nexus (Vicon Motion Systems; Denver, CO, USA). Kinematic data were processed in Visual3D (C-Motion; Germantown, MD, USA) and MATLAB (MathWorks; Natick, MA, USA).

Prior to walking on the treadmill (Bertec; Columbus, OH, USA), each participant walked overground at their self-selected pace. Walking velocity was estimated from the pelvis velocity of three trials. Each participant then walked on the treadmill at this speed for 2–5 min until they felt comfortable with the task, and the speed was adjusted (usually decreased) for some subjects per their request. Participants were instructed not to use the handrails, and they wore a harness attached to the ZeroG system (Aretech; Ashburn, VA, USA) for safety.

An ankle weight of approximately 10% of body weight up to a maximum of 12 lbs. was attached unilaterally to induce an adaptation to gait. Preliminary testing showed that 10% was adequate to cause changes without causing the participant to stop walking or trip. Details regarding treadmill speed and weight applied for each group are provided in Part 1 of this study (Damiano et al., 2017).

Data collection consisted of five treadmill trials: a 2 min baseline, 6 min with the ankle weight on the non-dominant leg, 2 min post-weight, 6 min with the ankle weight on the dominant leg, and 2 min post-weight. Leg dominance was determined in the CP cohort as the less affected limb, while in the TD group leg dominance was appropriated from self-reported hand dominance. Each participant took a short standing rest in the middle of each 6 min trial to reduce fatigue. Each of the five trials began standing at rest on the treadmill followed by 0.3 m/s^2^ acceleration to the chosen self-selected speed. The ankle weight was attached and removed between trials while the participant stood still. They were instructed to keep their feet flat on the ground before and after the weighted trials to prevent acclimatization the new condition.

### Data Analyses

Hip and knee angles were used to quantify changes in kinematics during different walking conditions. The kinematics for each stride (heel strike to heel strike) were time normalized to 101 samples (0–100% of gait cycle) using cubic spline interpolation. We examined the peak joint angle and total range of motion during each stride at both the hip and knee joint. A general linear mixed model was used to evaluate group difference in hip and knee joint kinematics with leg (dominant, non-dominant) as the within subjects factor and group (TD, CP) as the between subjects factor and *post hoc* tests as indicated. Statistical analysis was performed in Matlab software (The Mathworks, Natick, MA, USA). To quantify the gait variability we computed the standard deviation of each joint (hip and knee) angle at each normalized time point across all strides of each trial, and then averaged over the normalized stride to compute a single measure of variability for each subject and condition. Variability was examined for statistical differences with a mixed model with leg, joint, and condition (baseline, non-dominant weighted, post non-dominant weighted, dominant weighted, and post dominant weighted) as within subject factors and group as a between subject factor. *Post hoc* tests using Welch’s t-test to account for unequal variances were performed to assess differences in group means. We report effect size as the ratio of the sum of squares explained to the total sum of squares (η^2^) for ANOVA and as the ratio of difference in means to the pooled standard deviation (Cohen’s *d*) for t-tests.

**FIGURE 1:**
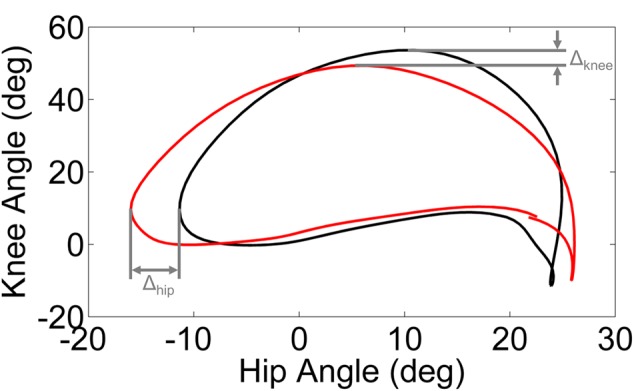
**Schematic illustration of Δ_Knee_ and Δ_Hip_ computed from the limit cycle (black line) and typical gait cycle phase (red line).** The limit cycle was computed as the mean phase of the final 95% of strides of the baseline walking trial.

Phase plots of hip angle vs. knee angle were examined for each leg (dominant/unaffected and non-dominant/affected) of every subject during each walking condition. Trajectory shifts in the phase space were used to quantify the adaptation in response to the perturbation and the after effects (i.e., unlearning or washout) following removal of the perturbation. We computed the limit cycle as the mean phase plot over the final 95% of the baseline trial. We utilized the displacement from the limit cycle at the point of maximum knee angle (Δ_Knee_) and minimum hip angle (Δ_Hip_) to quantify the phase shift between each walking condition and the baseline trial (**Figure 1**).

The question of whether kinematics across conditions differ by group is difficult to answer precisely because each condition (adaptation and post-adaptation) contains within it a transient period in which the kinematics of walking are changing, thus a general model testing for differences in group means may not be the best approach. One way of handling this, which has been applied in previous literature, is to split the adaptation and after-effects trials into early (e.g., first *x* strides) and late (e.g., last *x* strides) periods for purposes of comparison. However, setting the same early and late periods (i.e., the value of *x*) for each participant does not capture the underlying dynamics of the shifts in control in response to the perturbation, which was the goal here.

Instead, we performed a group analysis of Δ_Knee_ and Δ_Hip_ by plotting the group mean for each stride and fitting time constants to characterize convergence for each condition and group, as described below. Due to variation in the total number of strides across the group under each condition, the time course for this analysis was truncated to the minimum number for each cohort. The minimum number of gait cycles used to fit time constants was 67 and 88 for TD and CP groups, respectively, which required trimming a maximum of 42 and 48 gait cycles from each cohort. When plotted versus time, as measured by the stride number, these values provide a curve that quantifies convergence to the limit cycle. To parameterize this convergence, we fit an exponential function of the following form to each convergence curve:

$$\begin{array}{c} D(t)=K_{o}e^{\frac{t}{\tau }} \end{array}$$

where *D(t)* represents the distance from the limit cycle as a function of time *t* measured in strides, *K_o_* represents the initial distance from the limit cycle, and τ is the time constant that describes how quickly the walking converges. Note that τ can have a positive or negative sign, depending on the direction of the perturbation. The coefficients of the exponential function were estimated using iterative non-linear regression in Matlab. The regression procedure is moderately sensitive to the initial guess of parameter values. We used a t-statistic to evaluate the fit of the regression, and we adjusted the initial guess if *p* < 0.05 for any of the coefficients. For each condition and leg, we computed the time constant for group average convergence to baseline as a measure of adaptation. If we were unable to successfully fit a model to the group convergence after 100 repetitions, we did not report a time constant. The time constants fit to the unweighted trials immediately following the weighted trials were used to parameterize adaptation via the group after-effect.

Finally, we applied non-linear dynamical systems analysis to examine the effects of perturbation and adaptation on kinematics. Previous work has demonstrated that walking kinematics, when quantified using appropriate state variables, oscillate in a rhythmic but not strictly periodic manner, and thus, their closed loop trajectories constitute an attractor or limit cycle (Dingwell and Cusumano, 2000). We constructed multi-dimensional state spaces for each kinematic variable (left hip, right hip, left knee, and right knee) using a time embedding approach (Dingwell and Cusumano, 2000; Dingwell et al., 2001)

$$\begin{array}{c} S(t)=[\theta (t),\;\theta (t+\Delta t),\;\theta (t+2\Delta t),...,\theta (t+(d_{e}-1)\Delta t)] \end{array}$$

where *S(t)* is the *d_e_* dimensional state vector, *𝜃(t)* is the original kinematic time series, Δ*t* is the time delay, and *d_e_* is the embedding dimension. Similar to previous studies (Dingwell et al., 2001) Δ*t* was chosen to minimize correlation between components of the reconstructed vectors while *d_e_* was identified using a false nearest neighbors analysis. For our analysis Δ*t* = 10 samples (*f*_s_ = 120 Hz) and *d_e_* = 5, though it should be noted that dynamical system analyses have been shown to be insensitive to moderate changes in these parameters (Dingwell et al., 2007).

Local dynamic stability, a method for quantifying the effect of small perturbations during walking, was examined by fitting the average rates of divergence of initially neighboring trajectories as they evolved over each walking bout. In a similar manner as previous studies (Dingwell et al., 2001), we estimated local divergence exponents (λ) from the slope of a linear fit of the exponential divergence curve:

$$\begin{array}{c} y(i)=f_{s}*{\left\{\ln[d_{j}(i)]\right\}}_{\mu } \end{array}$$

where *y(i)* is the linear fit of the curve, *f_s_* is the sampling frequency, *d*_j_*(i)* is the Euclidean distance between the *j*th pair of initially nearest neighbors after *i* samples, and *{}_μ_* denotes the average over all *j* pairs. Short-term exponents (λ_s_) were calculated as the slope of the divergence between 0 and 1 stride, normalized by the average stride frequency. A larger value for λ_s_ indicates decreased stability.

## Results

### Kinematics

**FIGURE 2:**
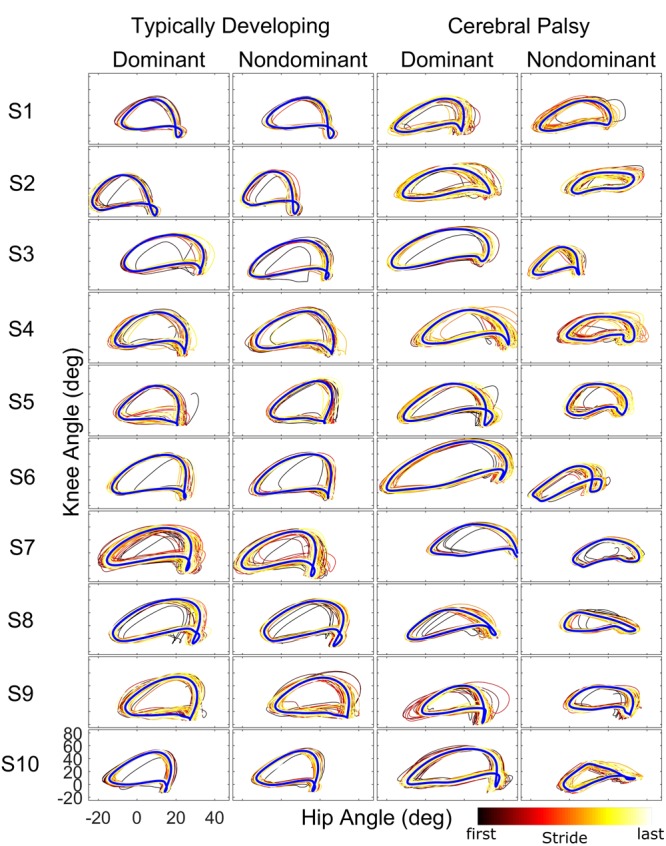
**Phase plots (hip angle vs. knee angle) during the baseline walking trial for the ten subjects of each cohort.** For each leg and subject, each stride of the baseline trial is depicted starting from the first stride (black) to the final stride (white) as indicated by the color bar. The blue line represents the limit cycle.

When examining kinematics at baseline, there was a noticeable asymmetry between the dominant and non-dominant legs in the group with CP (**Figure 2**). There was a significant interaction of group and leg on peak hip flexion during baseline (η^2^ = 0.10; *p* = 0.036), with *post hoc* tests indicating a significant effect for leg only in the CP group (*d* = 1.14; *p* = 0.020) with the dominant leg having a mean peak hip flexion of 35.8 ± 7.5° compared to 25.5 ± 10.3° for the non-dominant leg. There was a similar interaction at the knee (η^2^ = 0.14; *p* = 0.009), with no significant effect for leg dominance in TD but a significant effect in CP (*d* = 1.35; *p* = 0.008) with a mean peak knee flexion of 56.6 ± 12.4° in the dominant leg compared to 40.1 ± 12.0° in the non-dominant leg. For total hip excursion, analysis revealed no main or interaction effects for group or leg. At the knee, there was only a main effect for group (η^2^ = 0.43; *p* < 0.001) with the typically developing group having a mean of 65.1 ± 3.7° while the CP group had a mean of 50.9 ± 11.2°.

There were significant main effects on variability for joint (η^2^ = 0.26; *p* < 0.001) and condition (η^2^ = 0.06; *p* < 0.001). As expected, the knee joint kinematics showed significantly greater variability (mean 3.2°) than the hip joint (mean 2.2°). Likewise, variability was greater during the weighted trials than during baseline walking for the non-dominant (*d* = 0.60; *p* < 0.001) and dominant (*d* = 0.70; *p* < 0.001) limbs while variability in the two post-weight trials was not significantly different from baseline. There was a significant interaction effect on variability for group and leg (η^2^ = 0.03; *p* < 0.001). *Post hoc* tests showed a significant effect only in the CP group (*d* = 0.62; *p* < 0.001) with the dominant leg showing more variability than the non-dominant.

### Adaptation and After-effects

**FIGURE 3:**
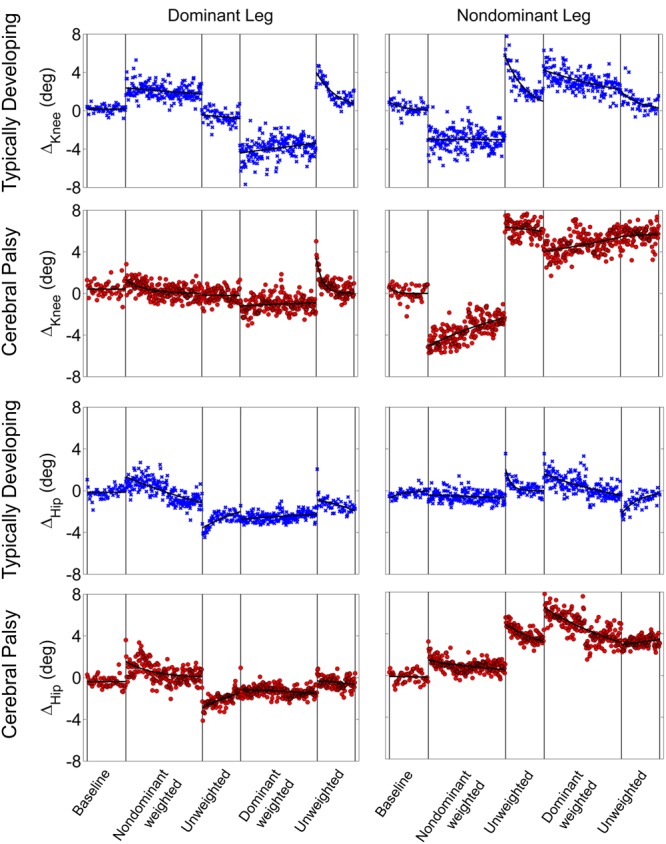
**Group mean changes in peak knee (Δ_Knee_) and peak hip (Δ_Hip_) across the experimental conditions for participants with cerebral palsy (red circle) and typical development (blue x).** Vertical lines indicate the transition between experimental conditions.

Stark differences were seen during adaptation to weighting in the knee and hip joints of the non-dominant leg between the CP and TD groups (**Figure 3**). The CP cohort showed more variability in peak knee (Δ_Knee_) and hip (Δ_Hip_) measures during walking than the TD cohort (**Table 1**). The largest variability was observed in the non-dominant leg of the CP group during the 2 min post-weight trial showing after-effects. At the knee, both groups showed an immediate knee flexion decrease in response to weighting the non-dominant limb, with the decrease initially larger in the CP group, but then slowly returning to baseline during the trial, while the TD remained roughly constant so that by the end of the weighted trials, the TD and CP groups had similar Δ_Knee_ values. In the group with CP, decreased knee flexion of the non-dominant leg during its weighting was accompanied by increased flexion of ipsilateral hip, a response not seen in the TD group. Interestingly, the contralateral (dominant) hip and knee flexion also increased immediately but transiently during that trial for both CP and TD groups. The non-dominant knee showed after-effects in both CP and TD, indicating that motor adaptation occurred in both groups. The TD group showed a relatively quick loss of the after effect while it persisted in the CP group as we were unable to fit a time constant (**Table 2**). However, the non-dominant hip showed an after-effect that disappeared in both groups, although the time constant was significantly slower in the CP group compared to TD. Interestingly, in the dominant leg, both TD and CP showed no after-effect in Δ_Knee_ and similar after-effect magnitudes and time constants for Δ_Hip_ during the unweighted period following the non-dominant weighting period, suggesting similar motor learning capability on the dominant side.

**Table 1:** Average standard deviation of peak knee (Δ_Knee_) and hip (Δ_Hip_) measures.

Condition	Δ_Hip_ (°)		Δ_Knee_ (°)	
	TD	CP	TD	CP
Baseline	1.23	3.78	1.75	4.83
Non-dominant weighted	1.86	3.83	3.18	4.13
Non-dominant unweighted	2.00	5.71	3.54	6.55
Dominant weighted	2.69	3.95	3.74	3.91
Dominant unweighted	2.99	3.99	2.91	3.96

**Table 2:** Time constants following unweighting.

		Ipsilateral		Contralateral	
		Δ_Knee_	Δ_Hip_	Δ_Knee_	Δ_Hip_
Typically developing	Dominant	57.7	–	–	172.1
	Non-dominant	56.2	13.7	51.7	47.5
Cerebral palsy	Dominant	16.6	–	–	147.8
	Non-dominant	–	239.1	–	–

*Time constants are reported in strides. Variables for which a model could not be accurately fit (as judged by t-statistic) are indicated by dash.*

Additionally, both TD and CP showed similar motor adaptation capability in the dominant limb in response to its weighting (**Figure 3**). At the knee an opposite pattern from the non-dominant side was observed across groups whereby the weight resulted in greater knee flexion reduction in TD than CP initially, a difference that slowly diminished such that Δ_Knee_ was similar across groups by the end of the weighted period. Remarkably, after-effects in the dominant knee disappeared more quickly in the CP group (**Table 2**), suggesting less adaptation in response to weighting. Response of the dominant hip to ipsilateral weighting was similar across groups; little after-effect was observed and a time constant was unable to be fit. As in the dominant leg, the non-dominant hip in both TD and CP showed immediately increased flexion in response to weighting the contralateral limb before returning toward baseline. However, in the CP group Δ_Hip_ returned only to the value reached at the end of the washout following the non-dominant weighted walking, and no after-effect nor time constant was observed while in TD Δ_Hip_ showed an after-effect that returned to baseline (**Table 2**). No after-effects for Δ_Knee_ were observed in the non-dominant leg following dominant weighting for either TD or CP. However, increased knee flexion was maintained in the CP group while knee flexion steadily returned toward baseline in the TD group.

### Stability

**FIGURE 4:**
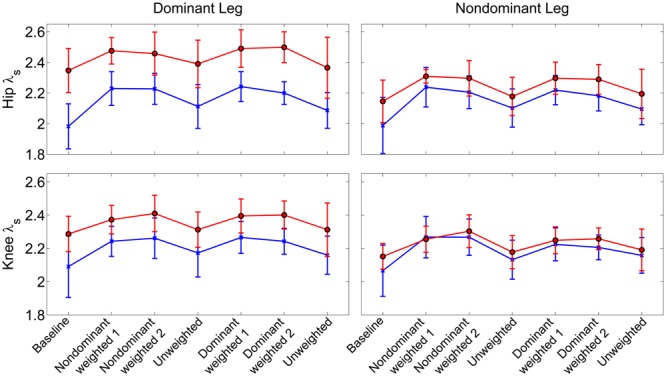
**Local stability as assessed by short term exponents across the experimental conditions at the knee and hip joints.** Exponents are plotted as group mean ± 1 standard deviation for each walking trial. Participants with cerebral palsy are shown in red, those with typical development are shown in blue. *Post hoc* testing showed significantly (*p* < 0.05) reduced stability in the hip and knee joint of the dominant limb of those with CP compared to TD.

Similar to joint excursion and variability, there was a pronounced baseline asymmetry in local stability in the group with CP which was not present in TD (**Figure 4**). At the hip, there was a significant main effect for condition (η^2^ = 0.16, *p* < 0.001), with both the non-dominant weighted (*d* = 1.12; *p* < 0.001; mean λ_s_ = 2.31 ± 0.14) and dominant weighted (*d* = 1.07; *p* < 0.001; mean λ_s_ = 2.30 ± 0.15) conditions showing reduced stability compared to baseline (mean λ_s_ = 2.11 ± 0.21). There were no significant differences between baseline and the trials after the weight was removed at the hip. There was an interaction between group and leg (η^2^ = 0.06; *p* < 0.001) with *post hoc* tests showing lower λ_s_ (*d* = 1.36; *p* < 0.001) in the non-dominant (2.24 ± 0.14) versus dominant hip (2.41 ± 0.14) in the CP group, indicating greater stability, while there were no differences between dominant and non-dominant legs in the TD group. Similar results were observed at the knee, with a significant main effect for condition (η^2^ = 0.16; *p* < 0.001) showing greater instability in the non-dominant (*d* = 1.14; *p* < 0.001; λ_s_ = 2.29 ± 0.11) and dominant (*d* = 1.04; *p* < 0.001; λ_s_ = 2.28 ± 0.11) weighted trials than baseline with no significant differences between baseline and unweighted. There was also a significant interaction between group and leg for the knee (η^2^ = 0.04; *p* < 0.001), with post-hoc tests showing significantly (*d* = 1.20; *p* < 0.001) less stability in the dominant leg (λ_s_ = 2.34 ± 0.13) compared to non-dominant leg (λ_s_ = 2.21 ± 0.13) for the group with CP and no significant differences across legs in controls. The increase in λ_s_ in the dominant leg accounted for the significant reduction in stability in those with CP compared to TD at the both the hip (*d* = 1.21; *p* < 0.001) and knee (*d* = 0.74; *p* < 0.001).

## Discussion

Our analysis showed that both children with hemiplegia from CP and typically developing children were able to adapt their intralimb kinematics in response to unilateral weighting. In the dominant limb, application of ∼10% body weight to the ankle resulted in similar adaptation and after-effect patterns in both groups, although children with CP displayed a washout period that was approximately three times faster than TD as judged by the time constant, a result that some may interpret as evidence that children with CP take longer to adapt walking patterns than TD. Another possible interpretation for this result is that children with CP have delayed motor development, given previous work showing that typically developing children younger than those in the age range of this study also take longer to adapt (Vasudevan et al., 2011). Yet, our analysis revealed asymmetry in the CP group with significantly greater peak knee flexion at baseline on the dominant side. The dominant side in this group also showed increased stride-to-stride knee angle variability in response to weighting compared to TD. Additionally, dominant knee and hip local dynamic stability was significantly reduced in CP compared to TD. While variability and local dynamic stability quantify fundamentally different aspects of locomotor behavior (Dingwell et al., 2001), the differences in each across groups suggest that the dominant leg is controlled differently in CP. Thus, it is not surprising that application of the same perturbation (by % body weight) may have a reduced effect on intralimb parameters such as knee angle in the stronger dominant leg. Given evidence that children with CP do adapt and show after-effects in the dominant leg (**Figure 2**), it may be possible that this perceived reduction in motor learning capacity (e.g., faster washout of after-effects) not be an indictment on the motor learning capability of those with CP, but instead may be attributed to a greater resistance to adapt due to the elevated functional role of the dominant limb in locomotion which makes it less susceptible to the perturbation. This possibility is further supported by the reduced effect of the weight on local stability in the dominant limb in CP compared to TD (**Figure 3**) and the fact that the ability of children with CP to adapt interlimb parameters, such as step lengths, was not different from TD (Damiano et al., 2017). Future studies utilizing a larger perturbation on the dominant limb could confirm this hypothesis.

In contrast to the dominant limb, we observed significant differences between CP and TD groups in the adaptation behavior, specifically the after-effects, of the non-dominant limb. In the TD group, non-dominant limb adaptation and after-effects of hip and knee kinematics in response to weighting were similar to their dominant limb. However, in CP the non-dominant leg showed a persistent after-effect which was not present in the dominant side or in the TD group, indicating that the CP group partly retained the adaptation on this side. Importantly, this result provides evidence that similar to healthy adults (Choi and Bastian, 2007), children with unilateral brain injury retain separate control circuits for each leg which can be adapted independently. Gait asymmetry in children with CP elucidated these distinct circuits which were not apparent in the symmetric TD group. Furthermore, we observed changes in the contralateral limb during unilateral weighting in both groups. Thus, coordination of neural control across legs, which has been previously demonstrated in healthy individuals (Ting et al., 2000; Verschueren et al., 2002) appears to be preserved in children with unilateral brain injury as well.

The persistence of adaptation after-effects in the non-dominant leg of the CP group may reveal an opportunity for potential therapeutic benefit. Similar to previous studies in adults with hemiplegia following stroke (Regnaux et al., 2008; Reisman et al., 2013) our results indicate that loading the more affected limb has beneficial effects. More specifically, when weighting the more affected (non-dominant) limb in the CP group, knee flexion – as judged by Δ_Knee_ – was significantly reduced followed by the after-effect of increased knee flexion by approximately 6° after weight removal. Given the initial 16° asymmetry in peak knee flexion at baseline, our results demonstrate that temporarily accentuating the error at the knee by unilateral weighting actually reduced the asymmetry when the perturbation was removed, at least during the short-term.

A similar effect is seen in individuals with hemispatial neglect following stroke, in which an optical deviation imposed via prism in the direction of the error results in a larger compensation than healthy controls (Rossetti et al., 1998). The resultant improvement in asymmetry was also found to be perturbation specific, as those with neglect did not adapt to a prismatic deviation in the direction opposite the functional error. Individuals with cerebellar damage have a reduced motor adaptation in response to similar prism perturbations (Martin et al., 1996), although it is not clear if adaptation is completely absent or proceeds more slowly. Interestingly, some individuals with cerebellar ataxia have diminished adaptation but a similar after-effect as controls (Fernandez-Ruiz et al., 2007). Conversely studies of individuals with basal ganglia disorders such as Huntington’s or Parkinson’s disease show similar capacity for adaptation to prisms but the after-effect is reduced (Fernandez-Ruiz et al., 2003). Taken together, these results demonstrate that visuomotor adaptation is a confluence of multiple neuronal processes that are differentially affected by neuropathology.

Similarly, motor adaptation in response to force perturbation is a result of multiple neural processes that adjust to the error and retain that information at different, i.e., slow and fast, rates (Smith et al., 2006). This hierarchy accounts for the observation in healthy individuals that the rate at which adaptation fades after removal of the perturbation is often faster than the initial adaptation. Our results show the same effect in both limbs of the TD group, but only on the dominant limb of the CP group. A complete return to baseline after weighting was non-existent, or at the very least much slower than the rate of adaptation in the more affected limb in those with CP. Retention of motor adaptation is more dependent on the slow component (i.e., 100s of trials) than the fast component (<10 trials) in healthy individuals (Joiner and Smith, 2008). In this study, both groups and both limbs experienced the perturbation for roughly the same time period (minimum of 198 gait cycles). Given the disparity in rate of de-adaptation, our results suggest that the sensitivity of this slow component is altered in the circuitry controlling the affected limb in children with unilateral brain injury. Indeed, neuroimaging studies have identified distinct neural circuitry activated during adaptation and de-adaptation to prisms (Chapman et al., 2010)and during slow and fast motor adaptation (Krakauer et al., 2004), supporting the possibility of this unilateral affect in response to a focal lesion. A limitation of this study was that our data were collected in a single session, and thus we are unable to assess whether this beneficial effect on the more affected limb was retained in the medium or long term; future studies will examine this possibility.

While stroke typically occurs in adulthood after walking has presumably become a refined motor skill, injury in CP happens during early development. On the one hand, this means that children with CP may not have an established motor repertoire to lean on post injury, yet on the other the potential for plasticity is reportedly greater in the developing central nervous system (Eyre, 2007), providing the opportunity for improved effectiveness of rehabilitation. This enhanced plasticity has been one pillar of hypotheses that robotic assisted training, which provides the opportunity for mass, task oriented practice under guidance of a controlled perturbation (i.e., robotic force), may expedite gait training outcomes (Dobkin and Duncan, 2012). While randomized controlled trials comparing robotic gait training to equal intensity therapies have not been performed in children with CP, studies in stroke survivors have shown no advantage (Hidler et al., 2009). Rather than using a robot to guide the user’s legs toward a specified gait pattern, adaptation via introduction of a perturbation may provide an improved training strategy. Recently, Wu et al. (2014), demonstrated that in healthy individuals, the motor control system restructures movement variability in such a way to promote learning in the perturbed environment. That is, following training with a specific perturbation, the motor system increases variability related to the perturbation, a process hypothesized to increase exploration in the task space, thereby potentiating skill learning. Here, we examined variability in hip and knee joint angle during walking, and found that variability increased during the weighted (training) trials in both TD and CP groups. *Post hoc* testing of the weighted trials showed no difference between limbs (dominant vs. non-dominant) in the TD group while variability increased more in the dominant limb compared to non-dominant during weighting in the CP group. This result suggests that elevated variability in response to weighting may actually expedite exploration of the task space, thus providing a potential mechanism to enable adaptation. Yet, the multi-rate models of motor adaptation described above suggest that extended exposure to the perturbation may be a more critical metric for retention of the adaptation (i.e., motor learning) than the amount of adaptation observed during the training session. Future studies should examine each of these factors to identify viable methods for training in this population.

In summary, this study demonstrated that children with hemiplegic CP are able to adapt intralimb kinematics in response to unilateral leg weighting during treadmill walking. The adaptation in the CP group was different than the healthy age-matched controls. The observed adaptation after-effects differed between the dominant and non-dominant sides in those with CP, suggesting that independent neural control of each limb is preserved in this population. Unilateral weighting of the more affected leg also resulted in a transient increase in symmetry, providing impetus to study this approach as a potential strategy for rehabilitation in these children.

## Author Contributions

TB, CS, and DD had substantial contributions to: the conception or design of the work; or the acquisition, analysis, or interpretation of data for the work; drafting the work or revising it critically for important intellectual content; and final approval of the version to be submitted for publication. All further agree to be accountable for all aspects of the work in ensuring that questions related to the accuracy or integrity of any part of the work are appropriately investigated and resolved.

## Conflict of Interest Statement

The authors declare that the research was conducted in the absence of any commercial or financial relationships that could be construed as a potential conflict of interest.
